# Microbial community structure and functional properties in permanently and seasonally flooded areas in Poyang Lake

**DOI:** 10.1038/s41598-020-61569-z

**Published:** 2020-03-16

**Authors:** Yang Liu, Ze Ren, Xiaodong Qu, Min Zhang, Yang Yu, Yuhang Zhang, Wenqi Peng

**Affiliations:** 1 State Key Laboratory of Simulation and Regulation of Water Cycle in River Basin, China Institute of Water Resources and Hydropower Research, Beijing, 100038 China; 2 Department of Water Environment, China Institute of Water Resources and Hydropower Research, Beijing, 100038 China; 3 Advanced Institute of Natural Sciences, Beijing Normal University, Zhuhai, 519085 China; 4 Flathead Lake Biological Station, University of Montana, Polson, MT 59860 USA

**Keywords:** Freshwater ecology, Microbial ecology

## Abstract

Water level fluctuations are an inherent feature regulating the ecological structures and functions of lakes. It is vital to understand the effects of water level fluctuations on bacterial communities and metabolic characteristics in freshwater lakes in a changing world. However, information on the microbial community structure and functional properties in permanently and seasonally flooded areas are lacking. Poyang Lake is a typical seasonal lake linked to the Yangtze River and is significantly affected by water level fluctuations. Bottom water was collected from 12 sampling sites: seven inundated for the whole year (inundated areas) and five drained during the dry season (emerged areas). High-throughput 16S rRNA gene sequencing was used to identify the bacterial communities. The results showed that the taxonomic structure and potential functions of the bacterial communities were significantly different between the inundated and emerged areas. *Cyanobacteria* was dominant in both areas, but the relative abundance of *Cyanobacteria* was much higher in the emerged areas than in the inundated areas. Bacterial communities were taxonomically sensitive in the inundated areas and functionally sensitive in the emerged areas. Nitrogen, phosphorus, and dissolved organic carbon concentrations and their ratios, as well as dissolved oxygen, played important roles in promoting the bacterial taxonomic and functional compositional patterns in both areas. According to the metabolic predictions based on 16S rRNA gene sequences, the relative abundance of functional genes related to assimilatory nitrate reduction in the emerged areas was higher than in the inundated areas, and the relative abundance of functional genes related to dissimilatory nitrate reduction in the inundated areas was higher. These differences might have been caused by the nitrogen differences between the permanently and seasonally flooded areas caused by intra-annual water level fluctuations. The relative abundance of functional genes associated with denitrification was not significantly different in the inundated and emerged areas. This study improved our knowledge of bacterial community structure and nitrogen metabolic processes in permanently and seasonally flooded areas caused by water level fluctuations in a seasonal lake.

## Introduction

Microbial assemblages are fundamental components of aquatic ecosystems and play important roles in driving global energy fluxes and biogeochemical cycling^1^. Microbial communities have high species diversity and genetic diversity^2–5^, which make them sensitive to environmental perturbations^6,7^. The relative balance of external nutrient loading influences microbial functional genes and associated metabolic processes in lake ecosystems^8^. Understanding the taxonomic and functional compositions of microbial communities is essential to elucidating the responses of natural bacterial communities to environmental perturbations, such as hydrological fluctuations and nutrient pollution^9–11^.

Poyang Lake (PYL) is a seasonal lake characterized by recurrent wet-dry phases^12–14^. Precipitation, evaporation, catchment inflows, and the Three Gorges Dam control the hydrological processes of PYL by regulating its water level^15^, which plays a crucial role in determining hydrodynamic processes^16^. In PYL, some low-lying areas (inundated areas) are inundated with water throughout the whole year, while some higher areas (emerged areas) are only inundated for a few weeks or months during the wet season^17,18^. During the dry season from October to March, the water level decreases and emerged areas dry. The water level increases in the wet season and the whole lake area is inundated^13,14^. During the wet season, some dissolved and particulate organic matter and nutrients are released from the sediment, affecting the chemical characteristics of the water bodies^19^. Previous studies have suggested that water level fluctuations could regulate the transformation of nutrients by changing bacterial activities in active erosion and transport zones^17,20–24^. In seasonal lakes, drying and inundation cycles within a year can influence nitrogen cycling by regulating nitrogen metabolism pathways^25–27^, such as anammox, nitrification, denitrification, and nitrogen fixation^28–30^.

Determining metabolic functions related to the nitrogen cycle of the microbial communities in seasonal lakes is essential for understanding their roles in biogeochemical processes related to nutrient cycles^2,3,31,32^ and their response to water level fluctuations^24,27,33,34^. Previous study indicated that bacterial communities are distinct taxonomically and functionally in the dry-season and wet-season in Poyang Lake^24^. Based on the previous study, we hypothesize that changing the water level may also affect the bacterial community composition in permanently and seasonally flooded areas in PYL. In this study, we compared the taxonomic composition and predicted functional traits (especially nitrogen metabolism pathways) of bacterial communities between permanently (inundated areas) and seasonally (emerged areas) flooded areas and explored their relationships with variations in environmental factors. Our aim was to study the differences in bacterial community structure and potential functions between permanently and seasonally flooded areas caused by water level fluctuations in PYL.

## Results

### Environmental variables

**Figure 1:**
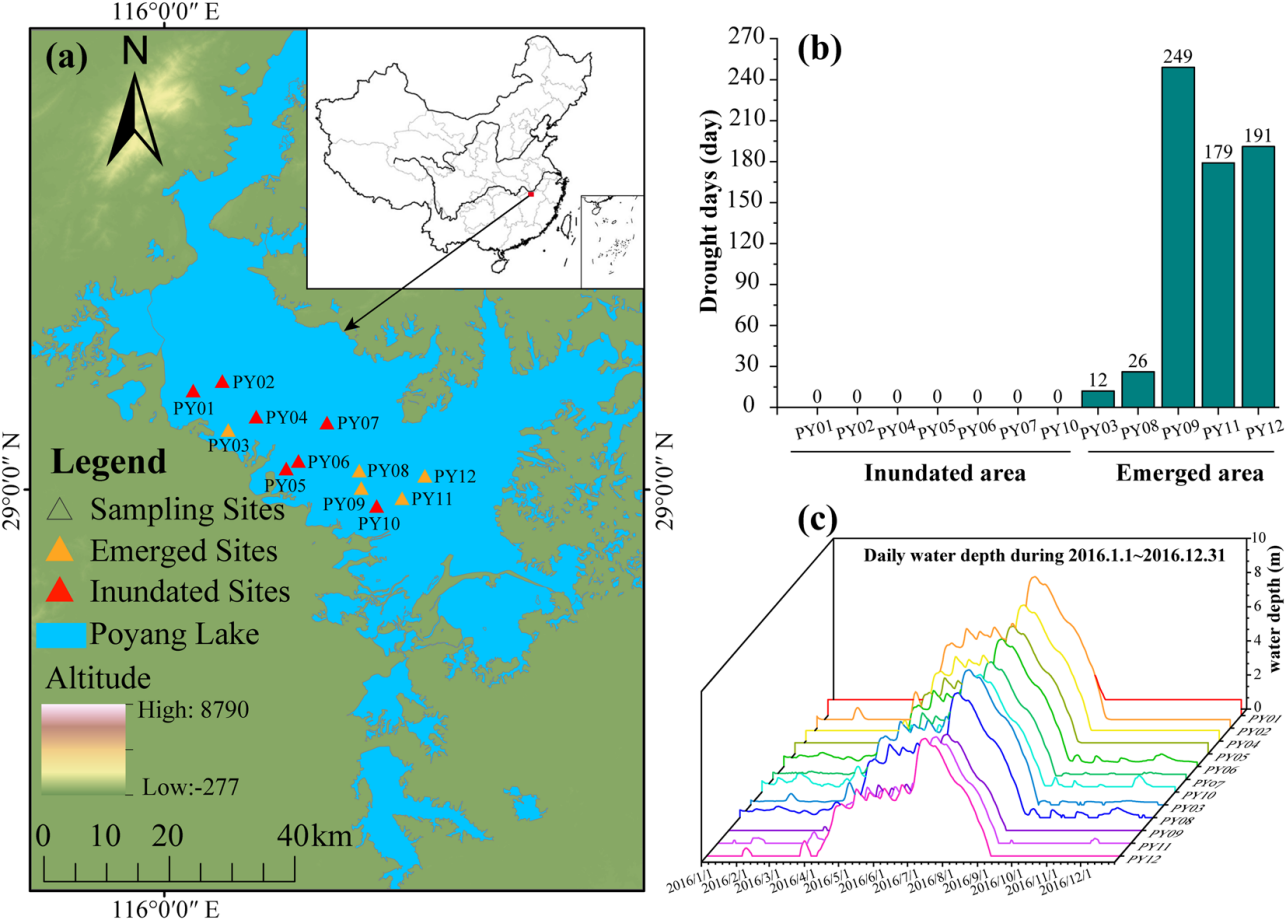
(**a**) Study area, (**b**) drought days at the sampling sites, and (**c**) daily water depth at the sampling sites in 2016.

The Environmental Fluid Dynamics Code (EFDC) was applied to simulate daily water depths at each sampling site in 2016, and the results are visualized in Fig. 1b,c. According to daily water depths, the sampling sites were divided into two groups: inundated areas (PY01, PY02, PY04, PY05, PY06, PY07, and PY10) and emerged areas (PY03, PY08, PY09, PY11, and PY12) (Fig. 1). Turbidity, total phosphorus (TP), phosphate (PO_4_^−^), and ammonium (NH_4_^+^) were significantly higher in the emerged areas than in the inundated areas (*p* < 0.05), while the C/P and N/P ratios were significantly higher in the inundated areas than in the emerged areas (Table 1, t-test, *p* < 0.05). Dissolved organic carbon (DOC) was not significantly different between the two areas (*p* > 0.05).

**Table 1:** Environmental variables in the inundated areas and emerged areas.

	Inundated areas		Emerged areas		*p* (t-test)
	Average	SD	Average	SD	
Temp (°C)	33.94	0.70	32.94	1.07	0.080
Salinity	1.54	0.57	1.54	0.29	0.992
Turbidity	9.49	9.41	18.14	12.30	0.050*
Transparent	1.42	0.44	1.63	0.39	0.420
pH	8.80	0.33	8.70	0.26	0.593
Cond	97.50	1.86	95.76	1.45	0.122
DO	5.13	0.68	5.13	0.33	0.993
DOC	2.09	0.21	2.10	0.33	0.949
TP	0.014	0.002	0.028	0.02	0.043*
PO_4_^−^	0.011	0.001	0.021	0.01	0.048*
TN	1.09	0.19	0.99	0.07	0.316
NO_3_^−^	0.88	0.11	0.83	0.08	0.418
NH_4_^+^	0.01	0.003	0.03	0.02	0.039*
C/N	3.25	0.36	3.58	0.67	0.305
C/P	401.62	59.91	275.32	131.40	0.042*
N/P	179.18	38.07	109.85	54.44	0.027*

Note: “*” indicates level of statistical significance at *p* < 0.05, “**” indicates *p* < 0.01. SD: standard division, Temp: Temperature, Cond: conductivity, DO: dissolved oxygen, DOC: dissolved organic carbon, TP: total phosphorus, and TN: total nitrogen.

### Bacterial community structure

**Figure 2:**
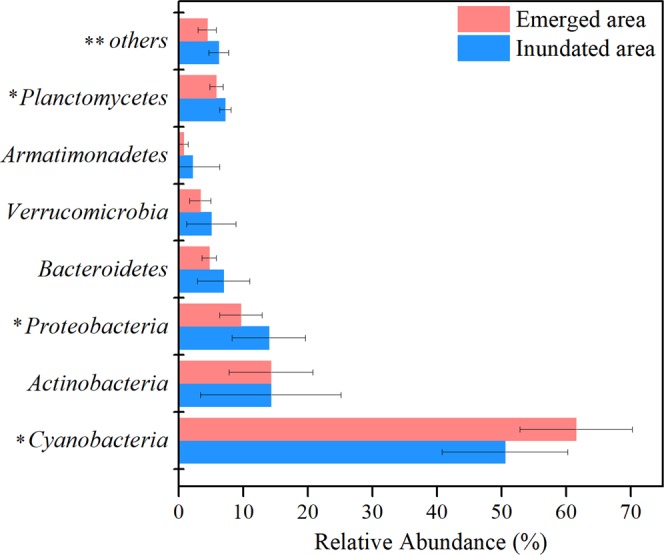
Relative abundances of bacterial phyla in the inundated areas and emerged areas. Only phyla with a relative abundance >1% in inundated and emerged areas are shown; “others” represent the unassigned operational taxonomic units (OTUs) and the phyla with a relative abundance <1%. The comparison of bacterial community composition in inundated and emerged areas was assessed using t-tests. “**” indicates levels of statistical significance at *p* < 0.01, and “*” indicates *p* < 0.05.

According to t-tests, the relative abundances of the phyla differed dramatically between the inundated and emerged areas (Fig. 2). The OTUs shared by the two groups were 61.6% of the total number of OTUs. The unique OTUs of the inundated and emerged areas was 17.23% and 21.17% of the total number of OTUs, respectively. In the inundated and emerged areas, the dominant phylum was *Cyanobacteria* (50.52% and 61.54%), followed by *Actinobacteria* (14.25% and 14.27%) and *Proteobacteria* (13.94% and 9.60%, respectively). The relative abundance of *Cyanobacteria* in the emerged areas was significantly higher than in the inundated areas (*p* < 0.05), while the relative abundances of *Proteobacteria* and *Planctomycetes* were significantly higher in the inundated areas (*p* < 0.05).

Heatmaps and analysis of similarity (ANOSIM) were conducted to reveal the differences of taxonomic composition and potential metabolic functions between bacterial communities in the two areas. A heatmap showed that the bacterial communities in the inundated areas were clustered apart from the communities in the emerged areas according to taxonomic composition, while there were no significant differences of potential metabolic functions between the two areas (Fig. S1). ANOSIM analysis showed that bacterial communities were significantly different in taxonomic composition between the two groups (*r* = 0.252, *p* < 0.05), while the potential functions were not significantly different (*r* = 0.087, *p* > 0.05). The differences in the taxonomic and functional alpha diversities were not significant between the two groups (Table S1, t-test, *p* > 0.05).

### Bacterial co-occurrence

**Figure 3:**
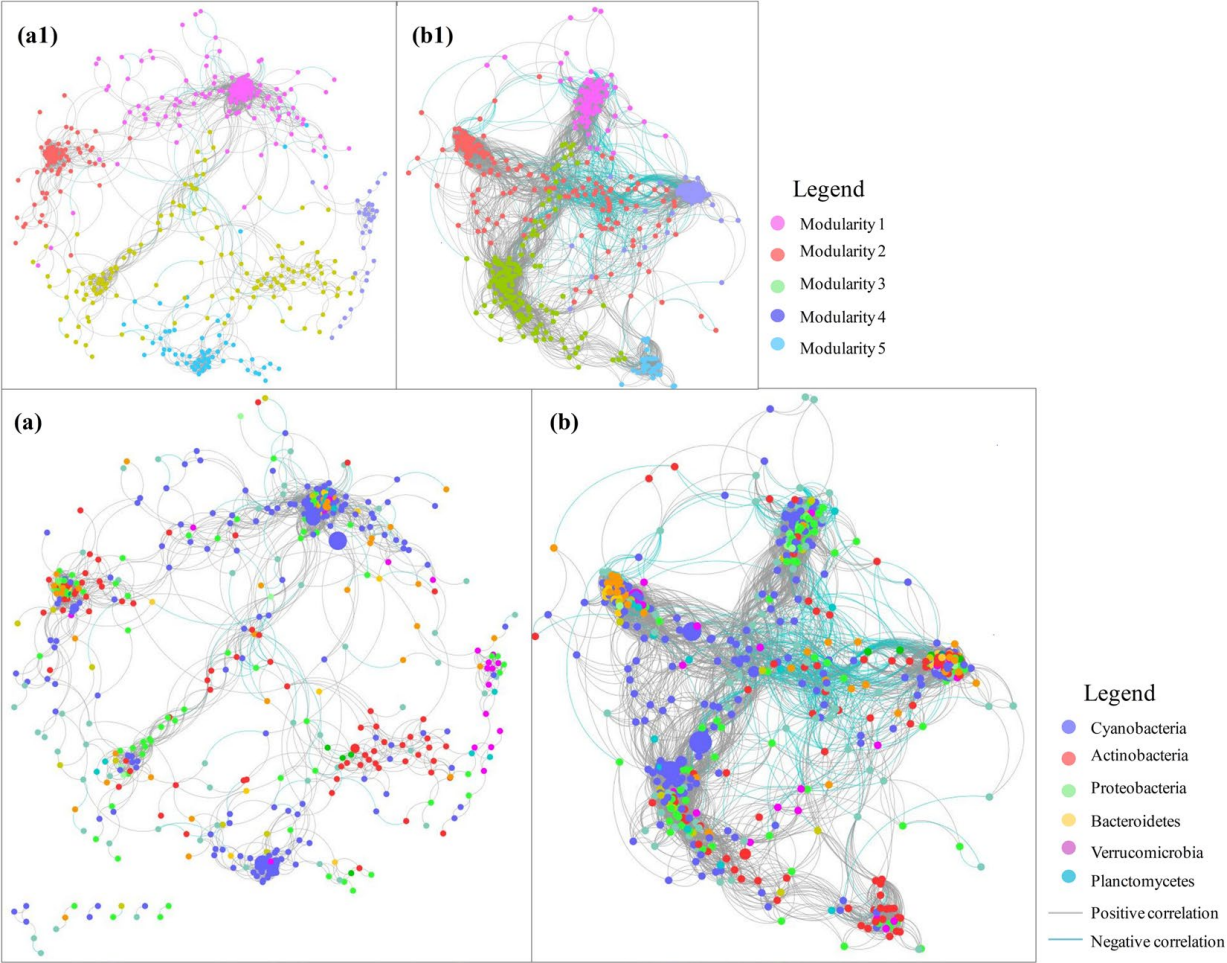
Co-occurrence networks of bacterial communities in the (**a**) emerged and (**b**) inundated areas. Modularity networks of (**a1**) emerged and (**b1**) inundated areas are shown. Circular nodes represent OTUs with a relative abundance higher than 0.01%. Edges represent Spearman’s correlations (Spearman’s *r* > 0.9 or *r* < −0.9, *p* < 0.05). The grey and blue lines indicate positive and negative correlations, respectively.

We constructed two networks based on samples from the inundated and emerged areas (Fig. 3) and calculated nine topological parameters to assess the interactions between the OTUs in the two networks (Table 2). The bacterial network for the inundated areas contained 500 nodes and 4218 edges (Fig. 3a), and the network for the emerged areas contained 477 nodes and 4158 edges (Fig. 3b). The proportions of positive OTUs correlations in the bacterial networks of the inundated and emerged areas were 97.7% and 98.20%, respectively. Network diameter, network centralization, network heterogeneity, and characteristic path length values were significantly higher in the emerged areas than in the inundated areas. Compared with the emerged areas, the network density, clustering coefficient, and modularity values were much higher in the inundated areas. Microbial assemblages in the two groups exhibited modular structures, and the modularity was notably higher in the inundated areas than in the emerged areas. Nodes with high degrees, high closeness centrality, and low betweenness centrality were considered keystone taxa. In the inundated areas, 40% of the top 10 keystone taxa were *Proteobacteria* and 20% were *Cyanobacteria*. In the emerged areas, *Proteobacteria* and *Cyanobacteria* represented 40% and 50% of the top 10 keystone taxa, respectively.

**Table 2:** Topological parameters for the microbial networks in the inundated areas and emerged areas.

Topological Parameter	Emerged areas	Inundated areas
Number of Nodes	477	500
Network Density	0.037	0.075
Network Diameter	18	8
Network Centralization	0.123	0.063
Network Heterogeneity	1.168	0.496
Characteristic Path Length	5.964	3.552
Clustering Coefficient	0.543	0.671
Modularity	0.633	0.754

### Potential functional properties

The Phylogenetic Investigation of Communities by Reconstruction of Unobserved States (PICRUSt) was used to predict the potential functions of the bacterial communities based on 16S rRNA sequences. According to the KEGG database, the potential metabolic functions were classified into Genes and Genomes orthologies (KOs) at three different pathway levels (Fig. S2). The nearest sequenced taxon index (NSTI) was determined for each sample to assess the accuracy of the functional prediction. The mean value of NSTI was 0.09 + 0.02 for all samples, indicating the high accuracy of the predicted metabolic functions in our study. To investigate the differences between the metabolic pathways related to core resources, genes associated with energy metabolism, amino acid metabolism, and carbohydrate metabolism (at level-3) were compared. The relative abundances of the functional genes associated with nitrogen metabolism were higher in the emerged areas than in the inundated areas.

**Figure 4:**
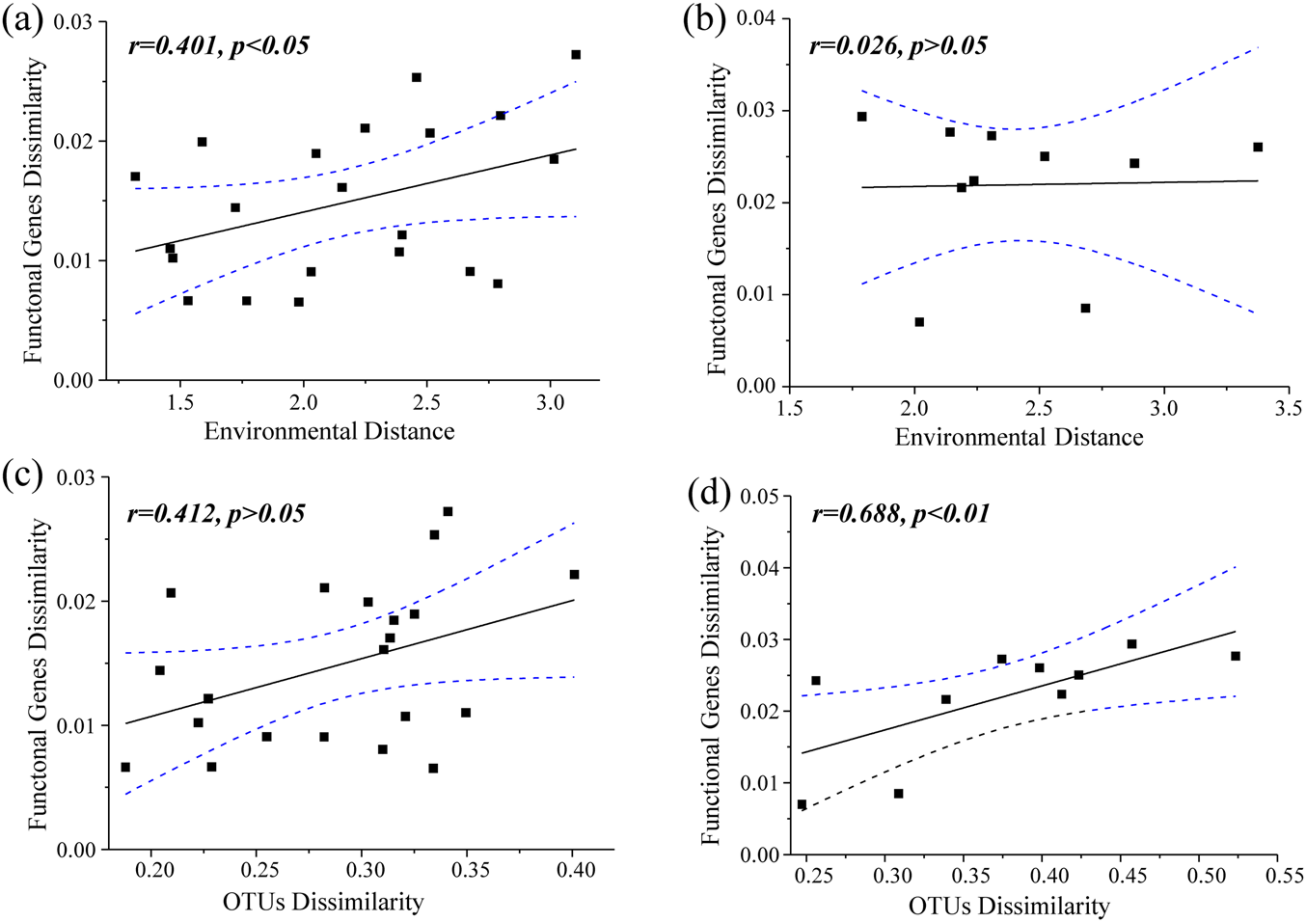
Mantel tests assessing the relationships between functional gene dissimilarity matrices based on Bray-Curtis distance and environmental distance matrices based on Euclidean distance in (**a**) emerged and (**b)** inundated areas and the relationships between functional and taxonomic composition of the bacterial community dissimilarity in (**c**) emerged and (**d**) inundated areas. One point represents one sample pair. The Pearson correlation coefficient (*r*) and statistical significance (*p*) of linear regression are shown. Blue dotted lines denote the 95% confidence interval.

**Figure 5:**
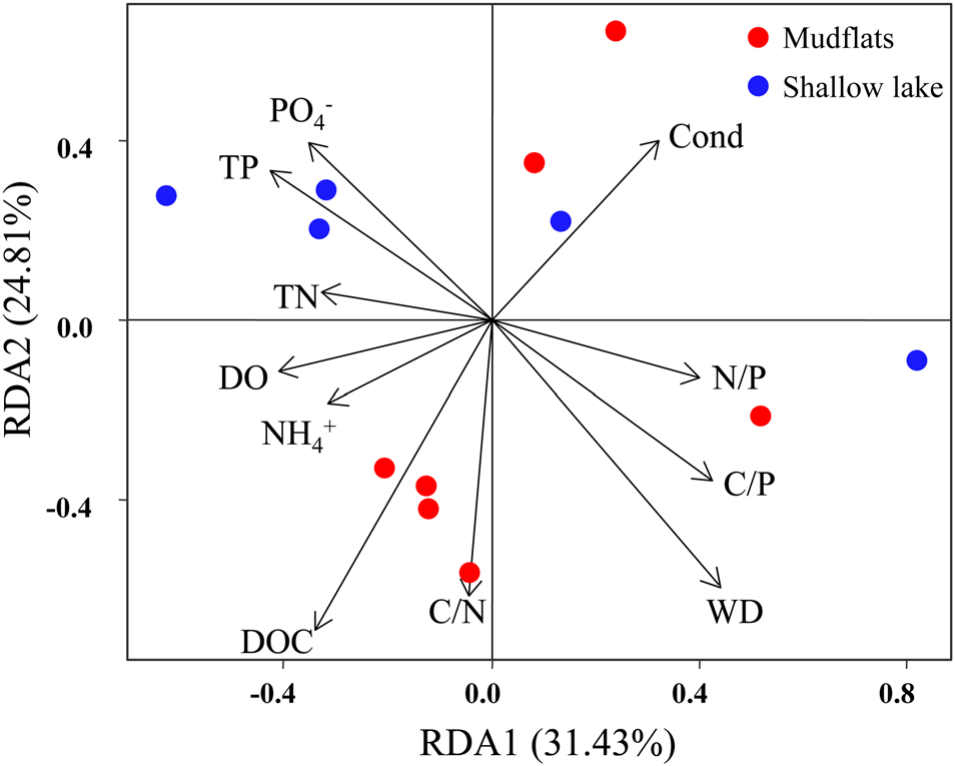
Redundancy analysis plots revealing the association of microbial communities and environmental factors. Only environmental factors that were significantly correlated with the microbial communities (Monte Carlo test, *p* < 0.05) are shown as solid black lines.

The correlations between the dissimilarities of the potential functions, the taxonomic composition of the bacterial community, and environmental factors were assessed by Mantel tests. There was a significantly positive relationship between potential functional genes’ dissimilarity and environmental distance in the emerged areas (Fig. 4a, *r* = 0.401, *p* < 0.05), while it was not significant in the inundated areas (Fig. 4b, *r* = 0.026, *p* > 0.05). Mantel tests revealed a significantly positive relationship between potential functional genes’ dissimilarity and taxonomic dissimilarity in the inundated areas (Fig. 4d*, r* = 0.688, *p* < 0.05), while was not significant in the emerged areas (Fig. 4c, *r* = 0.412, *p* > 0.05). Redundancy analysis (RDA) was applied to analyze the relationships between bacterial community distributions and environmental factors (Fig. 5). The first two axes accounted for 56.24% of the variance (RDA 1: 31.43%; RDA 2: 24.81%). According to the Monte Carlo test (*p* < 0.05), conductivity (Cond), dissolved oxygen (DO), water depth (WD), DOC, NH_4_^+^, TP, PO_4_^−^, C/N, C/P, and N/P were associated with bacterial community distribution of the two groups.

### Potential nitrogen metabolism

**Figure 6:**
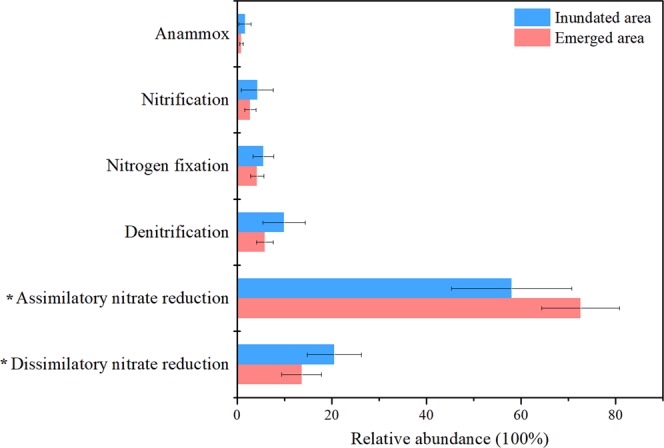
Relative abundances of the functional genes encoding the enzymes that catalyse nitrogen cycling pathways based on the KEGG database. “*” indicates *p* < 0.05 level.

We analyzed the relative abundance of the potential functional genes encoding pathways related to nitrogen cycling. The relative abundance of potential functional genes associated with assimilatory nitrate reduction (ANR) was significantly higher in the emerged areas than in the inundated areas, while the relative abundance of potential functional genes associated with dissimilatory nitrate reduction (DNR) was higher in the inundated areas (Fig. 6). We conducted Spearman correlations between the relative abundance of potential functional genes related to nitrogen metabolism pathways and environmental factors (Table 3). In the emerged areas, ANR was negatively correlated with pH and NO_3_^−^ but positively correlated with turbidity and NH_4_^+^ (p < 0.05). DNR was positively correlated with pH, TN, and NO_3_^−^ (p < 0.05). Potential denitrification was positively associated with TN, NO_3_^−^, and DOC. Potential nitrification was negatively correlated with turbidity. Moreover, potential anammox was negatively linked with TP (p < 0.05). In the inundated areas, DNR was positively correlated with TN, NO_3_^−^ and DOC. ANR was positively correlated with turbidity and negatively correlated with TN and DOC (p < 0.05). Potential denitrification was positively correlated with TN, NO_3_^−^, and DOC. Potential anammox was positively correlated with turbidity (p < 0.05).

**Table 3:** Spearman’s correlations between potential nitrogen metabolism processes and abiotic environmental factors in the inundated areas and emerged areas.

	WD	pH	Turbidity	TP	PO_4_^−^	TN	NO_3_^−^	NH_4_^+^	DOC
**Inundated areas**									
Dissimilatory nitrate	−0.536	0.564	−0.700	0.103	0.264	0.900*	0.700*	0.051	0.800*
Assimilatory nitrate	0.321	−0.410	0.900*	−0.359	0.105	−0.800*	−0.600	−0.205	−0.900*
Denitrification	−0.393	0.564	−0.700*	0.103	0.264	0.900*	0.700*	0.051	0.800*
Nitrogen fixation	−0.429	−0.616	−0.600	0.975**	−0.158	−0.200	−0.500	0.564	0.100
Nitrification	−0.429	0.103	−0.700*	0.564	0.632	0.600	0.200	0.205	0.500
Anammox	0.250	0.051	0.900*	−0.821	−0.264	−0.500	−0.100	−0.359	−0.600
**Emerged areas**									
Dissimilatory nitrate	0.303	0.677*	−0.392	−0.186	−0.163	0.566*	0.601*	−0.239	−0.084
Assimilatory nitrate	0.110	−0.635*	0.883*	0.193	0.200	−0.538	−0.622*	0.604*	0.112
Denitrification	0.324	0.510	−0.590*	−0.285	−0.152	0.601*	0.664*	−0.298	0.677*
Nitrogen fixation	−0.510	0.131	−0.119	0.271	0.138	−0.028	−0.056	0.070	−0.427
Nitrification	0.368	0.466	−0.580*	0.091	0.069	0.413	−0.336	0.004	0.028
Anammox	0.109	−0.109	0.287	−0.612*	−0.174	0.070	0.238	−0.421	−0.392

Note: “*” indicates level of statistical significance at *p* < 0.05, “**” indicates *p* < 0.01.

## Discussion

### Microbial assemblages and co-occurrence network

Small changes in water levels may have crucial effects on bacterial distribution and structure by influencing microhabitat conditions^21,35–37^. This study showed that *Cyanobacteria* was the dominant phylum in the inundated and emerged areas in PYL, while the relative abundance of *Cyanobacteria* was much higher in the emerged areas than in the inundated areas (Fig. 2). Previous studies have indicated that phosphorus and nitrogen are the main causes of freshwater eutrophication, and long-lasting *Cyanobacteria* blooms are tightly associated with nutrient dynamics^38–41^. In the inundated areas, dissolved phosphorus and nitrogen can be released into sedimentary pore water and lake water in a short time^18,21^ and used in microbial growth in the water^42^. In our study, the emerged areas had higher turbidity, NH_4_^+^, and TP than that of the inundated areas (Table 1), and these nutrients were crucial factors influencing the microbial assemblages in the studied areas (Fig. 5). Previous studies also have determined that these nutrients are the dominant factors influencing the structure and composition of microbial communities in erosion areas^43,44^.

Microbial co-occurrence patterns were analyzed to assess community assembly rules and interaction networks in highly complex systems^31,45,46^. Compared with that in the emerged areas, the bacterial co-occurrence network in the inundated areas was more complex, which could have been caused by the continuous hydrological connection in the inundated areas during the whole year^47,48^. Previous studies have also indicated that positive correlations between nodes in co-occurrence networks of desert soil bacterial communities could be the result of functional interdependencies among bacterial taxa under extreme environmental conditions^49–51^. In our study, high proportions of positive correlations between nodes in the two networks suggested the interdependencies among bacterial taxa under environmental disturbance. Topological parameters provide important information to help us to understand microbial community structure^2,3,47,52^. Higher values of network centralization and heterogeneity in the emerged areas than in the inundated areas suggested that there were many tightly connected bacterial modules (subnetworks in the whole network) in the emerged areas. Species interactions are more frequent and intense in a module than in the remainder of a community^47^. Thus, a minor disturbance in a main module of a bacterial network could have a large impact on the whole network of microbial communities. Moreover, higher mean degree and high closeness centrality, as well as lower betweenness centrality, could be used collectively to identify bacterial keystone taxa^53,54^, which were mainly classified as *Proteobacteria* and *Cyanobacteria* in the emerged and inundated areas, respectively. These keystone taxa could exert considerable influence on the structure and function of bacterial communities^54^. Removal of those strongly connected taxa in a network would cause the collapse of the freshwater ecosystem structure and function^55,56^.

### Potential functional genes and nitrogen metabolism

Seasonal water level fluctuations are one of the dominant forces controlling lake ecosystem functions, and any changes in water level could affect microbial metabolic functions^21,36,37,57^. To gain more insights into the effects of the inundated conditions on microbial functions, we calculated the relative abundance of potential functional genes related to metabolism pathways at level-1, level-2, and level-3 in the emerged and inundated areas. According to the previous studies^58–60^, the low NSTI mean values (0.09 + 0.02) indicated high accuracy of the predicted potential metabolic functions in our study. In general, the functional genes’ composition is strongly correlated with taxonomic composition in freshwater ecosystems^2,3,61–64^. In our study, taxonomic dissimilarity drove the potential metabolic functions of the bacterial communities in the inundated areas. Moreover, environmental variables were vital factors influencing the potential metabolic functions of the bacterial community in the emerged areas (Fig. 4). In the wet season, the re-flooding process in the emerged areas could cause the release of sedimentary nutrients^42^, and these nutrients might determine bacterial functional attributes^14,65,66^.

Metabolic functions related to the nitrogen cycle are particularly susceptible to environmental fluctuations because there are large differences in nitrogen metabolic processes with different forms of nitrogen^67,68^. Nitrogen in multiple chemical forms is cycled by a suite of coupled biogeochemical processes catalyzed by microbe-derived enzymes^1,69,70^. In our study, the relative abundances of the functional genes related to ANR were higher in the emerged areas, while the functional genes related to DNR were higher in the inundated areas (Fig. S2c). In general, ANR processes are much more prevalent than DNR processes in natural ecosystems^3,71^, and high concentrations of NH_4_^+^ could stimulate assimilatory pathways^67,71,72^. The reduction processes of NO_3_^−^ to NO_2_^−^ and then to NH_4_^+^ are part of the ANR processes, which usually occur in anaerobic environments such as sediment and soil^73^. In the wet season, a large amount of mineralized nitrogen (especially NH_4_^+^) could be easily dissolved in pore water and be rapidly released into lake water, and these mineralized nitrogen types could be used by bacterial communities as an energy source^3,71,74^. DNR is another nitrogen catabolic pathway that can retain nitrogen in the system in a bioavailable form for further biological processes^71,75^. Bacterial communities tend to use mineralized nitrogen as an energy source for growth in the emerged areas while as a nitrogen source for cell biosynthesis in the inundated areas. In addition, denitrification is another important pathway related to nitrogen metabolism. Through denitrification, nitrogen can be transformed into N_2_O or N_2_ and released into the atmosphere^71^. The addition of nitrate plus phosphate might affect microbial community structure, stimulate microbial enzyme activities, and promote denitrification rates^18^. Thus, during the wet season, reflooding processes could influence the nitrogen metabolism of bacterial communities by stimulating the release of sedimentary dissolved nitrogen and phosphate, which might promote the release of N_2_ and N_2_O over a short period in the emerged areas. The actual measurements of N_2_ and N_2_O at the sediment-water interface were important for understanding the nitrogen cycle driven by microorganisms under water level fluctuations. Although there was no data for N_2_ and N_2_O in our study now, the actual measurements will be considered for future research.

In our study, we explored the differences in bacterial communities and potential metabolic functions between the inundated and emerged areas during the wet season. The study of water level fluctuations in different seasons is crucial to elucidating the effects of inundation duration on microbial community structure in future studies. Metabolic functions were predicted from 16S rRNA sequencing data using the PICRUSt approach in our study. Although metagenomics reflects potential rather than actual functional capacity, our data offer a window into the poorly understood bacterial metabolism in seasonal lakes affected by water level fluctuations. The actual measurements of such functions will be considered in our future research.

## Conclusion

Water level fluctuations and duration of inundation could affect the microbial communities and their potential metabolic functions in Poyang Lake. *Cyanobacteria* dominated the bacterial communities in the inundated and emerged areas, while the relative abundance of *Cyanobacteria* was higher in the emerged areas than in the inundated areas. The redundancy analysis revealed that nitrogen, phosphorus, and carbon played important roles in driving taxonomic and functional genes’ composition of bacterial communities. The relative abundance of the functional genes related to potential assimilatory nitrate reduction (ANR) was higher in the emerged areas than in the inundated areas, while the relative abundance of functional genes related to potential dissimilatory nitrate reduction (DNR) was higher in the inundated areas. NH_4_^+^ and turbidity were the crucial factors promoting the potential ANR processes in the emerged areas. The variations in DOC might influence potential denitrification processes, which could impact the production of N_2_ and N_2_O. Overall, this study increased our knowledge of the impacts of water level fluctuations on bacterial communities in permanently and seasonally flooded areas of seasonal lakes.

## Methods

### Study area

Poyang Lake (115°47′–116°45′E, 28°22′–29°45′N) is China’s largest freshwater lake, and its catchment is located on the south bank of the middle and lower reaches of the Yangtze River^76^. Poyang Lake shows typical interlacing changes among water and land due to the influence of water level fluctuations^14,15^. During the dry season, there is only a narrow and meandering channel in PYL due to the decrease in the water level^66,77,78^. With water drawdown, many inundated and emerged areas emerge in the dry seasons^15,34^. During the wet season, many inundated areas are connected with emerged areas to form the whole lake due to the increase in the water level^2,13,14,66,76–78^.

### Sampling and physicochemical analyses

Bottom water was collected from inundated and emerged areas in Poyang Lake in August 2016, and the distance of each sampling site to sediment was the same (Table S2). The study area and sampling site distribution are shown on the map (Fig. 1). The map was created in ArcGIS 10.2 (http://desktop.arcgis.com/en/arcmap/) using ASTER GDEM data downloaded from the United States Geological Survey [ASTER GDEM is a product of the Ministry of Economy, Trade, and Industry (METI) and the National Aeronautics and Space Administration (NASA)] (Fig. 1a). The Environmental Fluid Dynamics Code (EFDC)^79^ was used to simulate the daily water depth of PYL in 2016 (Fig. 1b,c). A total of 600 ml of water was filtered through Whatman nylon membrane filters (pore size: 0.2 μm), and these membrane filters were immediately stored at −80 °C for subsequent DNA extraction. At each sample site, the water temperature (Temp), dissolved oxygen (DO), pH, turbidity, and conductivity (Cond) were measured *in situ* using a YSI Model 80 meter (Yellow Springs Instruments, Yellow Springs, Ohio, USA). Water depth (WD) was measured using a digital ultrasonic echosounder (Umwelt and Wissenschafts Technik, Mondsee, Austria). Water samples were acid fixed and transported to the laboratory at 4 °C for chemical analysis. Total nitrogen (TN) was analyzed by ion chromatography after persulfate oxidation. Nitrate (NO_3_^−^) was determined by ion chromatography. NH_4_^+^ was analyzed using the indophenol colorimetric method. TP and PO_4_^−^ were quantified using the ammonium molybdate method after oxidation. DOC was analyzed using a Shimadzu TOC Analyzer (TOC-VCPH, Shimadzu Scientific Instruments, Columbia, Maryland).

### DNA extraction, PCR, and sequencing

Bacterial 16S rRNA genes were analyzed to determine microbial community structures. Genomic DNA of microbial samples was extracted following the manufacturer protocols using the PowerSoil DNA Isolation Kit (MoBio, Carlsbad, CA, USA). The V3 to V4 regions of the 16S rRNA genes were amplified using primers 806R (GGACTACHVGGGTWTCTAAT) and 338F (ACTCCTACGGGAGGCAGCA) (Invitrogen, Vienna, Austria). Polymerase chain reaction (PCR) was performed using a thermal cycler (model 2720, ABI, USA) following standard procedures: 1 min hot start at 80 °C, 5 min of initial denaturation at 94 °C, followed by 30 cycles of denaturation at 94 °C for 30 s, followed by annealing at 52 °C for 30 s, and 90 s of extension at 72 °C, with a final extension step at 72 °C for 10 min. Amplified DNA samples were verified by 1.0% agarose gel electrophoresis with 1x TAE buffer and purified using the gel extraction kit (Qiagen, Hilden, Germany). The final sequencing process was run on a MiSeq sequencing platform (Illumina, USA).

### Sequence analysis and functional genes prediction

In total, 657,365 raw sequences (available from the National Center for Biotechnology Information database under the BioProject number PRJNA436872 and the accession number SRP133903) were processed using the QIIME pipeline^80^. The forward and reverse reads were merged and assigned to samples based on the barcodes and truncated by removing the barcode and primer sequences. Quality filtering on merged sequences was performed and sequences that did not meet the following criteria were discarded: sequence length <200 bps, no ambiguous bases, and mean quality score>20. The sequences were compared with a reference database (RDP Gold database) using the UCHIME algorithm to detect chimeric sequences^81^ that were removed in QIIME. After quality filtration, 261,458 reads were clustered into operational taxonomic units (OTUs) with a complete linkage algorithm at 97% sequence identity level, and representative sequences from each OTU were identified by the Greengenes database. Potential functions were predicted from 16S rRNA data using PICRUSt^59,82^. The normalized OTU table was obtained by dividing the OTU table counts by marker gene copy numbers to estimate the abundance of each OTU. After data normalization, we obtained 151,116 sequences in the normalized OTU table to predict the potential functional genes using the KEGG database. The nearest sequenced taxon index (NSTI), the average branch length separating OTUs in each sample from the reference genome^59^, was calculated to assess the accuracy of the functional prediction. The predicted functional genes were further classified into metabolic pathways at different levels (level 1–3).

### Statistical analysis

We compared environmental factors and the taxonomic profiles of microbial communities between the emerged and inundated areas. To determine whether the relative abundance of the phyla and functional genes were significantly different between the two areas, SPSS (Version 12.0) was applied to conduct bootstrap t-tests. We used heatmap (using Heatplus and Gplots packages in R version 3.5.1) and analysis of similarity (ANOSIM, PAST 3.0) to determine the differences in bacterial community compositions and potential metabolic functions between the two areas. Network analyses were conducted to reveal the co-occurrence patterns of the bacterial communities. OTUs with an average relative abundance higher than 0.01% and with a presence in more than half of the samples were used. The pairwise correlations between OTUs were calculated using the Spearman correlation in R (version 3.3.2 and Hmisc package 4.0–1), and *p*-values were adjusted using the Benjamin-Hochberg procedure. Only strong (Spearman’s *r* > 0.9 or *r* < −0.9 and *p* < 0.05) correlations were considered. The network was visualized using Cytoscape (version 3.6.1). An edge-weighted and spring-embedded network was applied to display the co-occurrence patterns of the OTUs. The topological and node/edge metrics were calculated to analyze the interactions between the nodes in the networks. The modular structural analysis of each network was conducted using ClusterMaker in Cytoscape. Redundancy analysis (RDA) was applied to analyze the spatial distribution of the bacterial communities with respect to various environmental factors using R (version 3.3.2 and Vegan package 2.4). Monte Carlo permutations (*p* < 0.05) were used to select a set of environmental factors that had significant effects on the microbial distribution. Environmental factors with high partial correlation coefficients (*r* > 0.5, *p* < 0.05) and variance inflation factors >20 were eliminated from the final RDA. Mantel tests were run to assess the correlations between the dissimilarities of the functional and taxonomic composition of the bacterial community based on Bray–Curtis distance and correlations between the dissimilarities of function (Bray–Curtis distance) and environmental factors (Euclidean distance). Correlation analyses were conducted to assess the relationships between metabolic pathways and abiotic factors using the Spearman correlation in SPSS software (Version 12.0).

### Ethical approval

This article does not contain any studies with human participants performed by any of the authors.

## Supplementary information

Supplementary information.
