# Supplementary material for: Microbial community structure and functional properties in permanently and seasonally flooded areas in Poyang Lake
Source: Sci Rep. 2020 Mar 16;10:4819. doi: 10.1038/s41598-020-61569-z (PMC7076011; doi:10.1038/s41598-020-61569-z)
Supplement: Supplementary file 1 — Supplementary information. [file 41598_2020_61569_MOESM1_ESM.docx]

**Microbial community structure and functional properties in permanently and seasonally flooded areas in Poyang Lake**

Yang Liu^a,b^, Ze Ren^c,d*^, Xiaodong Qu^a,b*^, Min Zhang^a,b^, Yang Yu^a,b^, Yuhang Zhang^a,b^, Wenqi Peng^a,b^

^a^ State Key Laboratory of Simulation and Regulation of Water Cycle in River Basin, China Institute of Water Resources and Hydropower Research, Beijing, 100038 China

^b^ Department of Water Environment, China Institute of Water Resources and Hydropower Research, Beijing, 100038 China

^c^ Advanced Institute of Natural Sciences, Beijing Normal University, Zhuhai, 519085 China

^d^ Flathead Lake Biological Station, University of Montana, Polson, MT, 59860 USA

***Corresponding Author:**

Ze Ren, Xiaodong Qu

Email: Ze.Ren@umontana.edu, quxiaodong@iwhr.com

Figure S1 Heatmap showing the differences of (a) taxonomic and (b) functional of microbial communities between the inundated area and emerged area based on Bray–Curtis distance.


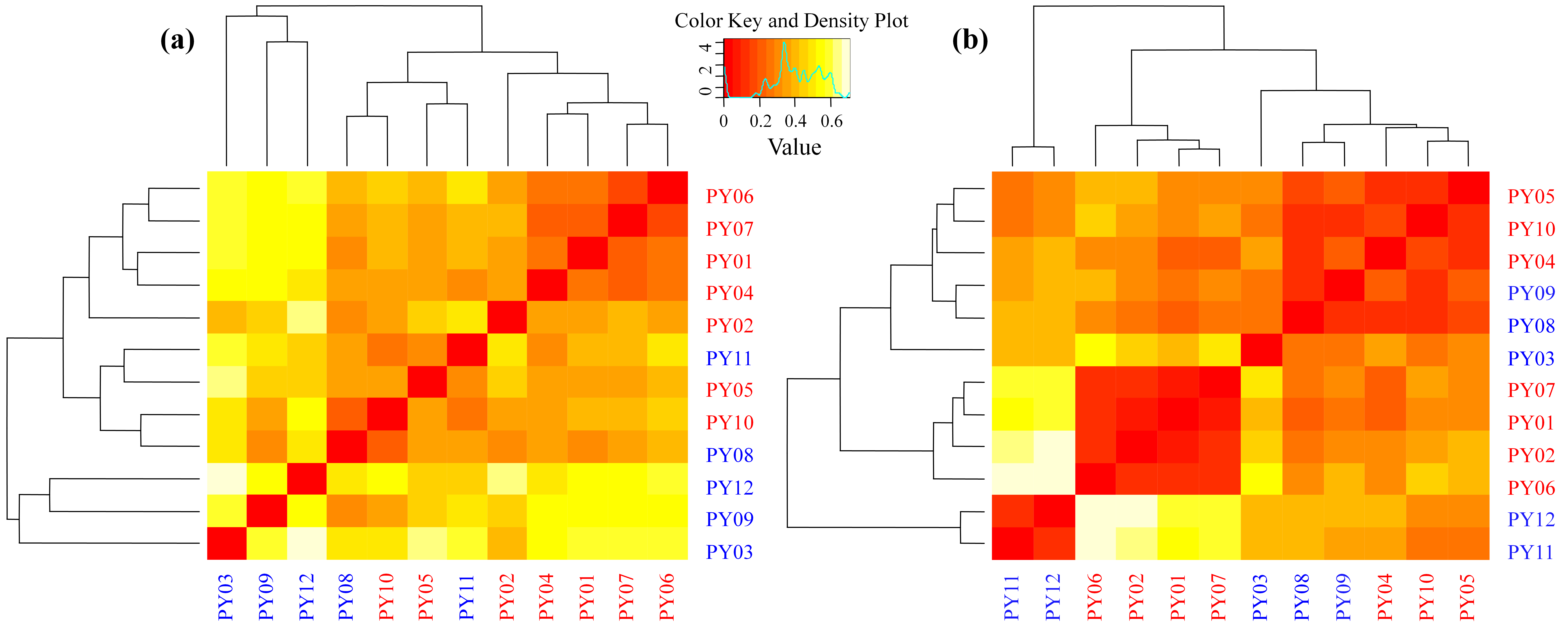


Figure S2 Relative abundances of various predicted function of microbial communities in the emerged area and inundated area using PICRUSt grouped into (a) level-1, (b) level-2, and (c) level-3 functional categories. “*” and “**” indicate *p*<0.05 level and *p*<0.01 level, respectively.


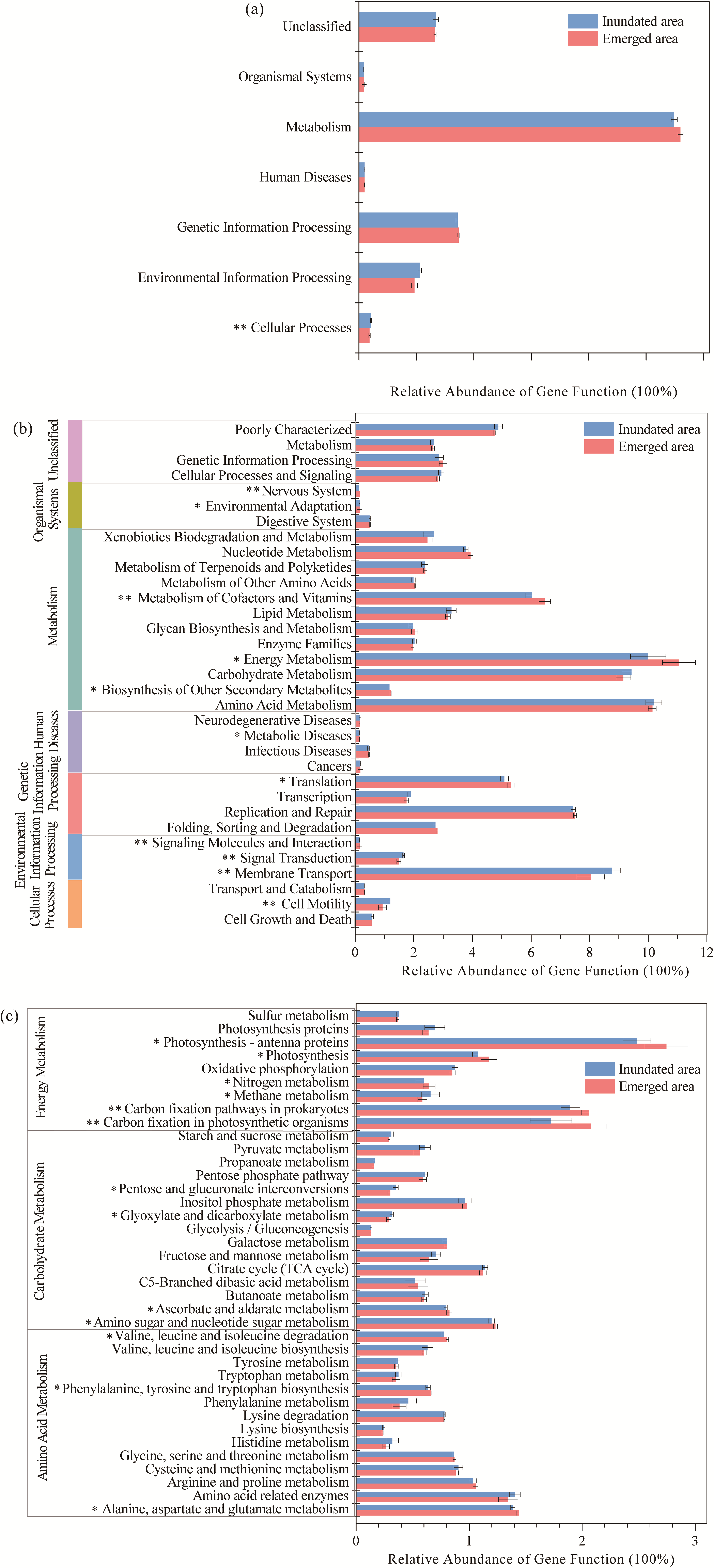


Table S1 Alpha diversity of microbial communities in the inundated area and emerged area in term of OTUs and functional genes.

|  | OTUs | | | Functional Genes | | |
| --- | --- | --- | --- | --- | --- | --- |
|  | Inundated area | Emerged area | *p*-value | Inundated area | Emerged area | *p*-value |
| Observed Species | 2050.20 | 2076.14 | 0.900 | 5225.80 | 5224.29 | 0.980 |
| Chao1 | 3349.52 | 3355.14 | 0.980 | 5354.01 | 5342.85 | 0.880 |
| Shannon | 7.72 | 7.16 | 0.221 | 5.35 | 4.96 | 0.221 |
| Simpson | 0.96 | 0.92 | 0.110 | 0.96 | 0.92 | 0.119 |

Table S2 Water depth of sampling sites.

| Group | Sampling sites | WD (m) | DSS (m) | DS (m) |
| --- | --- | --- | --- | --- |
| Inundated area | PY01 | 5.64 | 5.34 | 0.3 |
|  | PY02 | 7.04 | 6.74 | 0.3 |
|  | PY04 | 6.14 | 5.84 | 0.3 |
|  | PY05 | 6.07 | 5.77 | 0.3 |
|  | PY06 | 5.63 | 5.33 | 0.3 |
|  | PY07 | 5.47 | 5.17 | 0.3 |
|  | PY10 | 5.04 | 4.74 | 0.3 |
| Emerged area | PY03 | 4.66 | 4.36 | 0.3 |
|  | PY08 | 5.04 | 4.47 | 0.3 |
|  | PY09 | 3.79 | 3.49 | 0.3 |
|  | PY11 | 4.39 | 4.09 | 0.3 |
|  | PY12 | 4.84 | 4.54 | 0.3 |
| DW – water depth; DSS – depth of sampling sites; DS – distance of sampling sites to sediment | | | | |
